# TIMAP Upregulation Correlates Negatively with Survival in HER2- Negative Subtypes of Breast Cancer

**DOI:** 10.31557/APJCP.2021.22.6.1899

**Published:** 2021-06

**Authors:** Marya Obeidat, Khaldon Bodoor, Mohammad Alqudah, Amr Masaadeh, Marwa Barukba, Rowida Almomani

**Affiliations:** 1 *Department of Medical Laboratory Sciences, Jordan University of Science and Technology, Irbid, Jordan. *; 2 *Department of Applied Biology, Jordan University of Science and Technology, Irbid, Jordan. *; 3 *Department of Pathology, Jordan University of Science and Technology, Irbid, Jordan. *

**Keywords:** PPP1R16B, HER2, ER, PR

## Abstract

**Objective:**

*TIMAP* expression is regulated by transforming growth factor beta 1 (TGFβ1); known for its role in breast cancer development and metastasis. Nevertheless, data on TIMAP protein expression and its association with breast cancer development are lacking. In this study, we aimed to investigate the variation in TIMAP protein expression in breast cancer tissue and its correlation with various clinicopathological characteristics of breast cancer patients and overall survival rate.

**Methods:**

A total of 159 paraffin-embedded tissue blocks from women diagnosed with four breast cancer subtypes (49 HER2-only, 33 Luminal A, 39 Luminal B, and 38 triple negative) were used to construct tissue microarray (TMA), followed by TIMAP immunohistochemistry (IHC). *TIMAP* expression was scored by two pathologists and categorized as weak (1-33% expression), moderate (34-66%), and strong (67-100%). Chi-square test and Kaplan Meier survival test were performed to determine the association between *TIMAP* expression and clinicopathological features and overall survival rate, respectively.

**Results:**

TIMAP protein was strongly expressed in 46 (93.9%) HER2-only, 32 (97%) luminal A, 37 (94.9%) luminal B, and 29 (76.3%) triple negative. *TIMAP* expression negatively associated with *ER/PR* expression (P=0.03), and it negatively impacted the overall survival in HER2 negative group (P=0.02).

**Conclusion:**

Our findings suggest that TIMAP protein expression is upregulated in all breast cancer subtypes. However, its prognostic role is exclusively observed in HER2- negative group, suggesting a potential of targeting TIMAP in future therapeutic strategies in this group.

## Introduction

TIMAP (TGFβ-Inhibited Membrane-Associated Protein); or as annotated in GenBank PPP1R16B, is a regulatory subunit of protein phosphatase 1 catalytic subunit (PP1c) predominantly expressed in central nervous system (CNS) and hematopoietic cell lines (Cao et al., 2002; Magdaleno et al., 2002). It regulates PP1c enzymatic activity by targeting the holoenzyme to specific subcellular locations and substrates; subsequently affecting target signal transduction cascades involved in controlling various cell processes (Boratkó and Csortos, 2017). For example, it binds and regulates the phosphorylation state of the non-integrin laminin receptor1 (LAMR1) (Kim et al., 2005); best known for its association with tumor progression, metastasis (Fülöp and Larbi, 2002), and tumor angiogenesis (Iwamoto et al., 1996). In addition, TIMAP interacts with and regulates the function of many other key molecules involved in controlling cell adhesion, survival, proliferation and migration. Among these molecules are myosin light chain 2 (MLC2) (Shopik et al., 2013;Wang et al., 2019) and Ezrin- Radixin- Moesin (ERM) family (Csortos et al., 2008; Boratkó and Csortos, 2017) that stimulate dynamic remodeling of cell cytoskeleton during cell adhesion, proliferation and migration. Another example is the eukaryotic elongation factor-1A (eEF-1A) (Boratkó et al., 2015), which exhibits roles in cytoskeleton organization, apoptosis, and regulation of protein expression (Sasikumar et al., 2012). Moreover, TIMAP has been shown to regulate the phosphorylation level and subcellular localization of merlin (Boratkó et al., 2017); a tumor suppressor known for regulating cell proliferation (Morrison et al., 2001) and organizing cellular junctions (McClatchey and Fehon, 2009). Also, TIMAP inhibits the tumor suppressor (PTEN)-mediated suppression of Akt activity in human endothelial cells (Obeidat et al., 2014). Akt activation is key to promoting cell survival, proliferation, angiogenesis, and tumor progression. On the other hand, PTEN antagonizes Akt effects, and thus is inhibited in many tumors. Altogether, these studies suggest that TIMAP might play an important role in cancer. 

TIMAP is down regulated by transforming growth factor beta (TGFβ1) (Cao et al., 2002; Yang et al., 2017) which is well known for its role in regulating various cellular processes, including proliferation, apoptosis, migration, angiogenesis, differentiation, inflammation, and cell cytoskeletal remodeling (Clark and Coker, 1998). Furthermore, TGF-β is a key player in cancer development, and it is the main stimulator of epithelial-to-mesenchymal transition (EMT); a cellular process that potentiates cancer cell metastasis (Garcia et al., 2018). TGFβ is an important regulator of normal mammary gland development, as well as breast cancer development and progression (Burdette et al., 2005), where it plays a dual role (Huang et al., 2018); while in early stages of mammary carcinogenesis it acts as a tumor suppressor, in late stages it promotes tumor progression by potentiating tumor metastasis (Muraoka et al., 2002; Nam et al., 2008). Hence, it’s plausible to hypothesize that *TIMAP* expression might be altered in breast cancer.

Large scale mutagenesis analyses in mice sensitized to c-Myc-induced apoptosis identified TIMAP as one of key cellular oncogenes (Mendrysa et al., 2010). Moreover, according to RNA sequencing data, *TIMAP* expression appears to be altered in different solid tumors, including breast cancer, and hematological malignancies [https://www.proteinatlas.org/ENSG00000101445-PPP1R16B/pathology]. These data also indicated that it acts as a prognostic biomarker in head and neck and cervical cancers. However, to date, *TIMAP* expression at protein level and its role in breast cancer, or any kind of cancer for that matter, have not been studied. 

In the current study, we investigated TIMAP protein expression in 159 breast cancer tissue samples categorized into the following subtypes; Human epidermal growth factor receptor 2 (HER2)-only, Luminal A, Luminal B, and triple negative, by immunohistochemistry (IHC) using tissue microarray (TMA) and correlated its protein expression with different clinicopathological features and overall survival rate. 

## Materials and Methods

Tissue specimens. A total of 159 women diagnosed with various breast cancer subtypes were identified via retrospective analysis of the pathological records from the department of pathology at King Abdullah University Hospital (Irbid, Jordan). The patients had been treated with radical mastectomy for the tumor or axillary lymph node resection between the years 2007 and 2019. This study was approved by the Faculty of Medicine Research Ethics Committee at Jordan University of Science and Technology (Irbid, Jordan).

TMA and IHC. Archived paraffin-embedded breast carcinoma tissue blocks were used to construct the TMA using TMA Master II instrument (3DHISTECH Ltd., Budapest, Hungary). TMA tissue blocks were sectioned at 4 μm thickness and collected on Superfrost plus glass slides for processing by IHC using the BenchMark ULTRA system (Roche Diagnostics, Risch-Rotkreuz, Switzerland) as previously described (Bodoor et al., 2018; Bodoor et al., 2020). Rabbit polyclonal antibody against TIMAP/PPP1R16B (MyBioSource, Inc, San Diego, USA) at 1:200 dilution was used to determine TIMAP protein expression. The slides were scored as described previously (Bodoor et al., 2018; Bodoor et al., 2020), whereas slides with 1-33% expression were scored as weak and were considered negative in statistical analysis; slides with 34-66% expression were scored as moderate and were considered positive; and slides with 67-100% expression were scored as strong and were also considered as positive in statistical analysis. The expression of Estrogen receptor (ER), Progesterone receptor (PR) and HER2 was retrieved from the patients archived records. Tumor volume was calculated differently according to available data for dimensions; For one- dimension x: volume = 4/3*π*(x/2)3; for two dimensions x and y whereas x<y: volume = x2*y; and for three dimensions x, y and z: volume = x*y*z.

Statistical Analysis. The relationship between *TIMAP* expression level and clinicopathological characteristics was established by Chi-square test. One-sided Fisher’s exact test was considered more reliable for 2×2 crosstabs and thus was reported instead of Chi-square test where appropriate. Survival outcomes, defined as the period from time of diagnosis to death from any cause or the last contact, were estimated with the Kaplan Meier analysis and compared between groups by log-rank test. Statistical analyses were conducted using IBM SPSS Statistics for IOS, Version 26 (IBM Corp, Armonk, NY, USA). p-value ≤0.05 was considered significant. 

## Results

**Figure 1:**
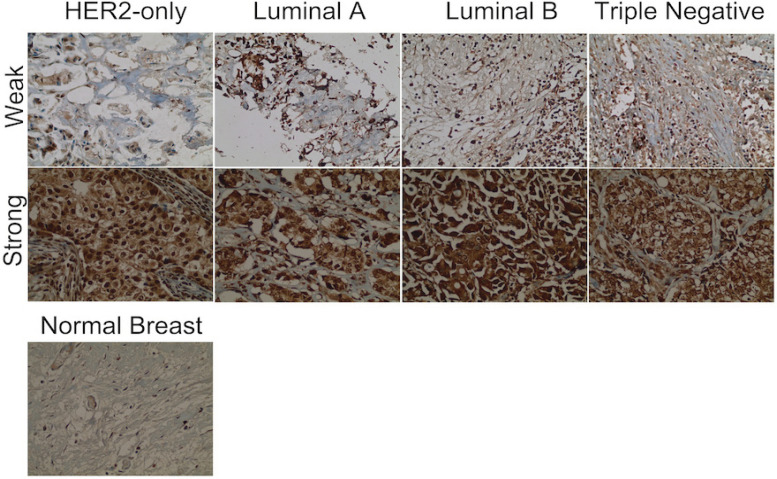
Immunohistochemical Images of *TIMAP *Expression in Breast Cancer Subtypes at 40X Magnification. Weak represents a negative expression and strong represents a positive expression. Normal breast tissue is a negative control

**Figure 2:**
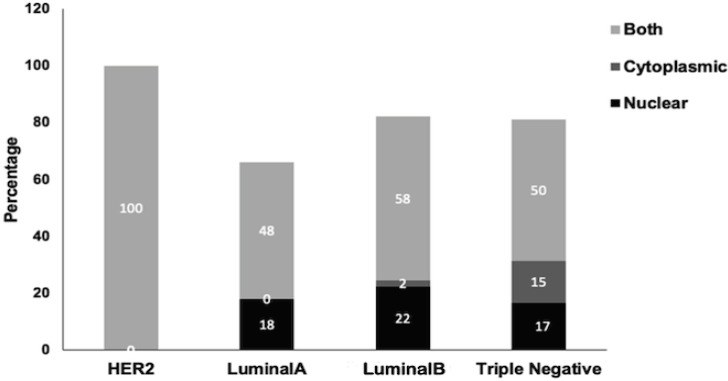
Percentage of Breast Cancer Samples Expressing *TIMAP* in Nucleus, Cytoplasm or Both

Patients clinicopathological characteristics and TIMAP expression levels. The clinicopathological characteristics of the 159 study subjects are shown in Table 1. Specimens of four molecular subtypes of breast cancer were included in the study; 49 HER2-only (ER-/ PR-/HER2+), 33 luminal A (ER+/PR+/HER2-), 39 luminal B (ER+/PR+/HER2+), and 38 triple negative (ER-/PR-/HER2-). *TIMAP* expression was scored according to percentage of positive cells (as described in the methods section), and it was categorized into “negative” for both null and weak expression; and “positive” for both moderate and strong expression (Figure 1). Positive *TIMAP* expression was found in 46 (93.9%) HER2-only subtype, 32 (97%) luminal A subtype, 37 (94.9%) luminal B subtype, 29 (76.3%) triple negative subtype (Table 2). 78.6% of samples exhibited both cytoplasmic and nuclear expression of *TIMAP*, while 17% and 4.4% had a dominant nuclear and cytoplasmic expression, respectively (Table 2 and Figure 2). 

Associations between *TIMAP* expression and clinicopathological characteristics of patients. Analyses of association between *TIMAP* expression and clinicopathological features of patients are displayed in Table 2. There was no significant association with most of the features. However, significant associations were observed between *TIMAP* expression and the molecular subtypes of breast cancer (p=0.007), *ER/PR* expression (P=0.03), and TIMAP subcellular localization (P=0.000). It is worth mentioning that there was a positive association between *TIMAP* expression and *HER2* expression (61 cases in HER2 negative group and 83 in HER2 positive), however, it did not reach statistical significance (P=0.06), while *TIMAP* expression negatively associated with *ER/PR* expression (75 cases in ER/PR negative group and 69 in ER/PR positive, P=0.03). 

**Figure 3:**
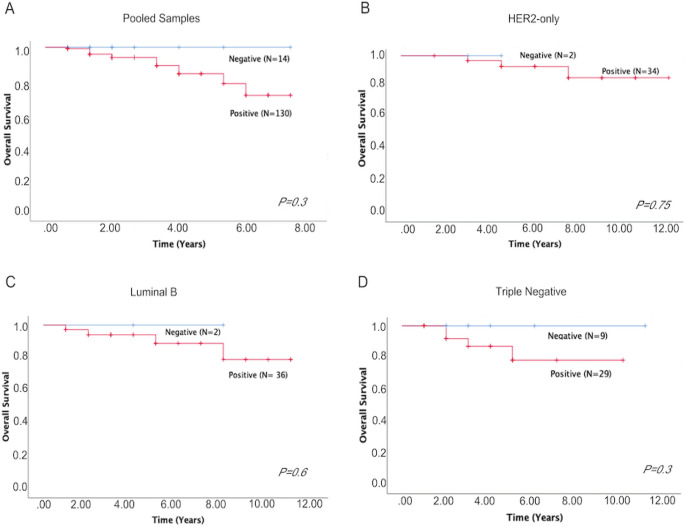
Overall Survival Analysis Based on *TIMAP* Expression in Pooled Samples and Different Breast Cancer Subtypes. A). Overall survival based on TIMAP expression in pooled samples. B). Overall survival based on TIMAP expression in HER2-only. C). Overall survival based on TIMAP expression in Luminal B. D). Overall survival based on TIMAP expression in triple negative

**Figure 4:**
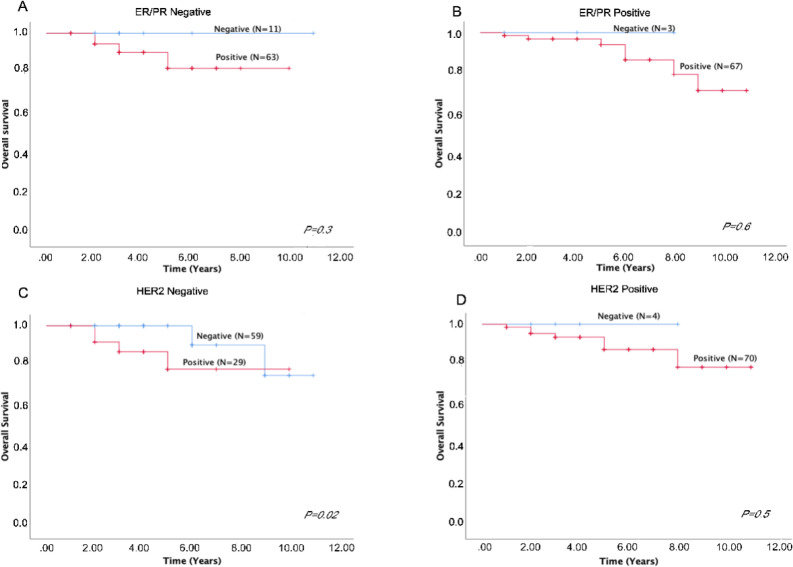
Overall Survival Analysis Based on *TIMAP* Expression in ER/PR and HER2 Negative and Positive Groups. A). Overall survival based on TIMAP expression in ER/PR negative group. B). Overall survival based on TIMAP expression in ER/PR positive groups. C). Overall survival based on TIMAP expression in HER2 negative groups. Overall survival based on TIMAP expression in HER2 positive groups

Associations between *TIMAP* expression and survival outcomes. We next investigated the impact of *TIMAP* expression on overall survival. There was no significant difference between negative and positive *TIMAP *expression when all subtypes were pooled together (Figure 3A, P=0.3), as well as when each subtype was analysed independently (Figure 3B (HER2- only); P=0.75, 3C (Luminal B); P=0.6, 3D (Triple negative); P=0.3). However, since *TIMAP* expression was negatively and positively correlated with *ER/PR* and *HER2* expression, respectively (Table 2), we further investigated its impact on overall survival in those groups. While there was no significant difference between negative and positive *TIMAP* expression among ER/PR negative (Figure 4A, P=0.3), ER/PR positive (Figure 4B, P=0.6), and HER2 positive groups (Figure 4C, P=0.5); there was a negative impact of* TIMAP* expression on overall survival in HER2 negative group (Figure 4D, P=0.02).

**Table 1:** Clinicopathological Characteristics of Breast Cancer Patients Included in This Study (n=159).

Characteristics	Group	N	%
Age	Mean (Range)	51.3 (28-82)	
	</=50	78	49.1
	>50	81	50.9
Histologic Grade	I, II	49	31.2
	III	108	68.8
Tumor Size	T1	8	5.1
	T2	82	52.2
	T3	49	31.2
	T4	18	11.5
Lymph Node Status	N0	40	27.6
	N1	30	20.7
	N2	31	21.4
	N3	44	30.3
Metastasis	M0	74	55.2
	M1	60	44.8
Tumor Stage	I	4	3.1
	II	40	30.8
	III	26	20
	IV	60	46.2
Axillary Lymph	Negative	39	26.7
	Positive	107	73.3
Vascular Invasion	Negative	32	25.6
	Positive	93	74.4
Family History	Negative	57	60.0
	Positive	38	40.0
Molecular Subtypes	HER2-only	49	30.8
	Luminal A	33	20.8
	Luminal B	39	24.5
	Triple Negative	38	23.9
Histologic Types	IDC	125	86.2
	Basal/like	6	4.1
	Metaplastic	3	2.1
	Medullary	6	4.1
	Micropapillary	5	3.4
DCIS	Absent	21	17.9
	Present	96	82.1
Treatment	Chemotherapy	39	33.1
	Adjuvant Chemotherapy	76	64.4
	Hormone Therapy	3	2.5

IDC, Invasive Ductal carcinoma; DCIS, Ductal Carcinoma In Situ.

**Table 2:** Association between *TIMAP *Expression and Clinicopathological Features of Breast Cancer Patients

Characteristic	Group	Negative	Positive	Total	P value
Age	≤ 50	8	69	78	0.4
	> 50	1	80	81	
Molecular Subtypes	HER2-only	3	46	49	0.007*
	Luminal A	1	32	33	
	Luminal B	2	37	39	
	Triple Negative	9	29	38	
ER/PR	ER-/PR-	12	75	87	0.03*
	ER+/PR+	3	69	72	
HER2	Negative	10	61	71	0.06
	Positive	5	83	88	
Histologic Grade	I	0	8	8	0.3
	II	2	39	41	
	III	12	96	108	
Tumor Size	T1	0	8	8	0.3
	T2	7	75	82	
	T3	5	44	49	
	T4	2	16	18	
lymph Node Status	N0	6	34	40	0.4
	N1	2	28	30	
	N2	4	27	31	
	N3	3	41	44	
Metastasis	M0	8	66	74	0.6
	M1	4	56	60	
Tumor Stage	I	0	4	4	0.8
	II	5	35	40	
	III	3	23	26	
	IV	4	56	60	
Axillary Lymph	Negative	5	34	39	0.7
	Positive	9	98	107	
Vascular Invasion	Negative	3	29	32	0.5
	Positive	7	86	93	
Family history	Negative	4	53	57	0.9
	Positive	4	34	38	
DCIS	Absent	2	19	21	0.9
	Present	10	86	96	
Histologic Types	IDC	9	116	125	
	Basal/like	1	5	6	0.4
	Metaplastic	0	3	3	
	Medullary	1	5	6	
	Micropapillary	1	4	5	
Location	Nuclear	3	24	27	0.000*
	Cytoplasmic	4	3	7	
	Both	8	117	125	
Treatment	Chemotherapy	4	35	39	0.64
	Adjuvant Chemotherapy	5	73	78	
	Hormone Therapy	0	2	2	

* p-value≤0.05 was considered significant.

## Discussion

Previous transcriptome studies of different cancer cohorts have shown that TIMAP is upregulated in various tumor types, whereas it acts as a prognostic marker in both cervical and head and neck cancers [https://www.proteinatlas.org/ENSG00000101445-PPP1R16B/pathology]. However, to date, immunohistochemical data of *TIMAP* expression in cancer tissue and its clinical significance are lacking. Here, we present a novel evidence of TIMAP protein expression in breast cancer tissue.

In our study, *TIMAP* expression was significantly augmented in all subtypes of breast cancer compared to normal breast tissue, which showed minimal to no* TIMAP* expression (Figure 1). This result is consistent with previous studies that demonstrated a hematopoietic and central nervous system (CNS)- predominant expression of *TIMAP* in normal tissue (Cao et al., 2002; Magdaleno et al., 2002), and that its expression, at least at transcript level, was upregulated in cancer [https://www.proteinatlas.org/ENSG00000101445-PPP1R16B/pathology]. However, our study provides an unprecedented evidence of *TIMA*P upregulated expression at protein level in breast cancer. 

Among the four subtypes studied here, *TIMA*P expression was observed less frequently in triple negative group, which is usually correlated with poor prognosis, while HER2-only, luminal A, and luminal B groups showed relatively similar and higher levels of expression. This result could not be directly related to the lack of expression of *ER, PR*, or *HER2*, as the non-triple negative subtypes express at least one of these proteins and show similar expression of *TIMAP*. However, since TIMAP has been shown to be downregulated by TGFβ1 (Cao et al., 2002; Yang et al., 2017) and that TGFβ1 is significantly upregulated in triple negative breast cancer as opposed to non-triple negative subtypes (Ding et al., 2016), it is plausible to suggest that the reduced level of TIMAP protein observed in the triple negative subtype here could be explained by presumably upregulated* TGFβ1 *expression. Nevertheless, future analyses are required to examine this possibility.

Furthermore, *TIMAP* expression was positively correlated with *HER2* expression and negatively correlated with *ER/PR* expression. Whether there is a regulatory relationship between these molecules is still unknown and requires further investigation. Nonetheless, since, overall, the prognosis of ER/PR-negative tumors is relatively poorer than for ER/PR-positive, targeting TIMAP may present a new therapeutic opportunity in treatment of challenging subtypes of breast cancer. 

Most breast cancer samples in this study, regardless of their subtypes, displayed both cytoplasmic and nuclear expression patterns of *TIMAP*. This is in agreement with previous studies that showed TIMAP can be localized to the plasma membrane, cytoplasm and the nucleus (Cao et al., 2002; Kim et al., 2005; Li et al., 2007; Csortos et al., 2008; Boratkó et al., 2015; Boratkó and Csortos, 2017); and this is attributed to its N-terminal nuclear localization signal and the C-terminal CAAX domain (Cao et al., 2002) that allow TIMAP to shuttle between these cell compartments.

It has been previously shown that overexpression of *TIMAP* induces endothelial cell survival, proliferation and angiogenesis (Obeidat et al., 2014), as well as TGFβ1- stimulated macrophage migration (Yang et al., 2017). Since these cellular processes are indispensable to tumor development and metastasis, we further analyzed whether* TIMAP* expression level was correlated with tumor development stage and metastasis state, however there was no significant correlation. 

Our survival analysis demonstrated a clear negative impact of *TIMAP* expression in HER2-negative groups that are usually more resistant to treatment. This finding further highlights the potential role of TIMAP as a therapeutic target and, thus, we recommend future investigations to address the molecular mechanism of this protein in breast cancer development and the possibility of targeting it in the treatment of this disease, especially in drug resistant subtypes.

## Author Contribution Statement

Marya Obeidat carried out the study design, examined the IHC staining, performed statistical analyses, drafted and prepared the final manuscript. Khaldon Bodoor participated in study design and helped in drafting and reviewing the manuscript. Mohammad Alqudah coordinated data collection and TMA and IHC staining, and pathological review of sections. Amro Masaadeh and Marwa Barukba scored the IHC staining and captured the microscopic images. Rowida Almomani participated in study design.
